# Admission route and use of invasive procedures during hospitalization for acute myocardial infarction: analysis of 2007-2011 National Health Insurance database

**DOI:** 10.4178/epih/e2015022

**Published:** 2015-05-01

**Authors:** Hyungseon Yeom, Dae Ryong Kang, Seong Kyung Cho, Seung Won Lee, Dong-Ho Shin, Hyeon Chang Kim

**Affiliations:** 1 Department of Preventive Medicine, Yonsei University College of Medicine, Seoul, Korea; 2 Research Affairs, Yonsei University College of Medicine, Seoul, Korea; 3 Division of Environmental Health, Environmental Policy Research Group, Seoul, Korea; 4 Department of Public Health, Yonsei University Graduate School, Seoul, Korea; 5 Severance Cardiovascular Hospital, Yonsei University College of Medicine, Seoul, Korea; 6 Cardiovascular and Metabolic Diseases Etiology Research Center, Yonsei University College of Medicine, Seoul, Korea

**Keywords:** Myocardial infarction, Hospitalization, Emergency departments, Vascular surgical procedures, Trends

## Abstract

**OBJECTIVES:**

The goal of this study was to investigate trends in admission to the emergency department and the use of invasive or surgical procedures during hospitalization for acute myocardial infarction (AMI) in Korea.

**METHODS:**

The National Health Insurance (NHI) claims database between 2007 and 2011 was used. We identified all admission claims that included codes from the tenth revision of the International Classification of Diseases beginning with I21 as the primary or secondary diagnosis. Information about the admission route, admission date, discharge date, and whether coronary artery angiography, angioplasty, or bypass surgery was performed was also obtained from the NHI database.

**RESULTS:**

Of the 513,886 relevant admission claims over the five years encompassed by this study, 295,001 discrete episodes of admission for AMI were identified by analyzing the date and length of each admission and the interval between admissions. The number of AMI admissions gradually decreased from 66,883 in 2007 to 47,656 in 2011. The number and proportion of admissions through the emergency department also decreased from 38,118 (57.0%) in 2007 to 24,001 (50.4%) in 2011. However, during the same period, admissions in which invasive or surgical procedures were performed increased from 15,342 (22.9%) to 17,505 (36.7%).

**CONCLUSIONS:**

The reported numbers of emergency department visits and admissions for AMI decreased from 2007 to 2011. However, only a small portion of the patients underwent invasive or surgical procedures during hospitalization, although the number of admissions involving invasive or surgical procedures has increased. These findings suggest that simply counting the number of admission claims cannot provide valid information on trends in AMI occurrence.

## INTRODUCTION

Acute myocardial infarction (AMI) is one of the leading causes of death worldwide [1,2]. According to National Health Insurance (NHI) claims data, medical service uses and costs associated with AMI in Korea have continuously increased over the last few decades [3]. The NHI claims data have been used to assess disease burden, because their database encompasses almost all medical usage in Korea under the mandatory universal health insurance system [4]. The NHI claims data have also been used to investigate disease prevalence and incidence [5-7]. However, since compensation for medical services is the main purpose of NHI claims, the NHI claims data do not include the information necessary for estimating disease frequency. For example, diagnoses in the NHI claim database do not perfectly match clinical diagnoses [8]. Moreover, multiple claims can be issued for a single disease event [5,6,9]. Therefore, it is necessary to identify and validate disease episodes in order to estimate disease frequency correctly [5-7].

Since early revascularization has been proven to improve the clinical outcome of AMI patients, the revascularization rate has increased in many countries, including Korea [10,11]. Shortening the initiation time for revascularization is also associated with improved outcomes in AMI patients [12]. Studies have reported that using emergency medical services shortened the initiation time for revascularization compared to patients who self-transported to a hospital [13,14]. However, no nationally representative study has investigated the utilization of medical resources by AMI patients. In particular, previous research has not addressed how many patients are admitted through the emergency department and how many patients undergo revascularization procedures during hospitalization. Most previous studies of AMI patients in Korea were limited to selected large hospitals, and only included subjects who were admitted through the emergency department [7,15].

Thus, we used the NHI claims database from 2007 to 2011 to investigate trends in AMI admissions through the emergency department and in AMI admissions involving invasive revascularization procedures.

## MATERIALS AND METHODS

We analyzed entries in the NHI claims database between January 1, 2007 and December 31, 2011. The database includes date of admission, date of discharge, admission route, diagnosis codes according to the tenth revision of the International Classification of Diseases, and details about which medical services were used during hospitalization [16].

**Figure 1.**
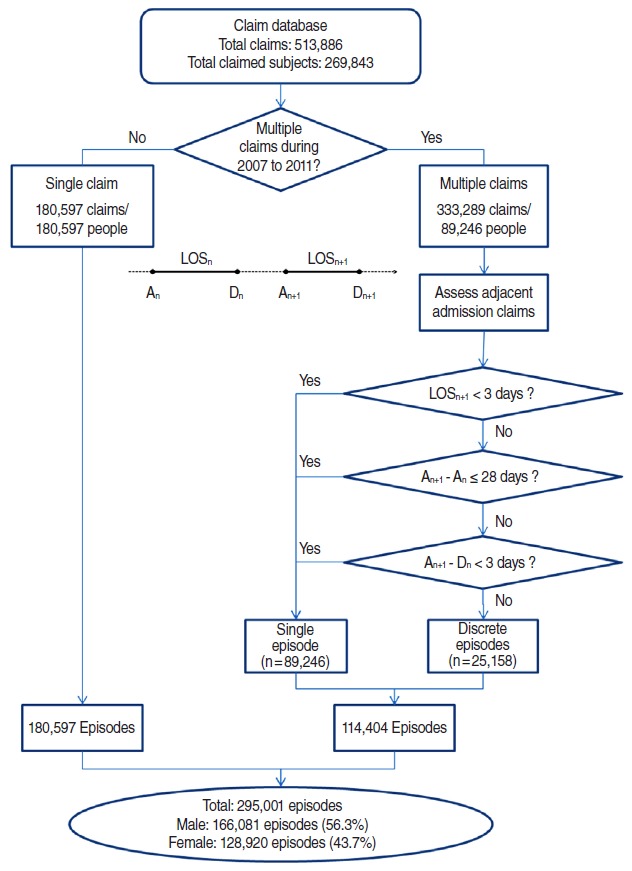
Algorithm for identifying admission episodes of acute myocardial infarction based on claims for acute myocardial infarction. A_n_, admission date of the n^th^ hospitalization; D_n_, discharge date of the n^th^ hospitalization; LOS^n^, length of stay of the nth hospitalization.

We aimed to assess how many patients with a diagnosis of AMI used emergency services and/or underwent invasive revascularization procedures, rather than attempting to estimate the absolute number of patients with AMI. Thus, we defined an AMI admission as a hospitalization claim with I21.X as the primary or secondary diagnosis. We did not include claims with the diagnosis codes I22.X– I25.X, because they represent secondary conditions after an AMI event or other ischemic heart diseases. We identified a total of 513,886 claims for AMI admissions among 269,843 patients during the five years of the study. Multiple claims can be issued for a single AMI episode, for instance, when a patient is admitted to more than one hospital, or admitted twice or more to the same hospital. Under the Korean NHI system, hospitals submit their claims for inpatient service to the Health Insurance Review and Assessment Service monthly. Therefore, multiple claims can be submitted for a single episode, if the hospitalization duration is especially long or happens to encompass multiple reporting periods. Thus, we analyzed the date of admission, date of discharge, and length of hospitalization in order to identify discrete AMI episodes. Two adjacent admission claims were regarded as the same episode of AMI when they met at least one of the following three conditions: 1) the admission date of the second hospitalization was within 28 days of the admission date of the first hospitalization; 2) the admission date of the second hospitalization was within three days of the discharge date of the first hospitalization; or 3) the length of the second hospitalization was less than three days (Figure 1) [9].

We assessed whether the hospitalized patient was admitted through the emergency department. In our analysis, invasive procedures included both diagnostic and therapeutic procedures involving the coronary arteries. Invasive therapeutic procedures included surgical and non-surgical revascularization techniques involving the coronary arteries, such as percutaneous transluminal coronary angioplasty and coronary artery bypass surgery. Coronary angiography, which is a diagnostic procedure, was also included because it is routinely performed to evaluate coronary artery occlusion and is frequently accompanied by therapeutic procedures.

## RESULTS

We initially identified 513,886 claims from 269,843 patients for hospitalization with a diagnosis code of I21.X between 2007 and 2011. These figures included 180,597 patients with a single claim and 89,246 patients with multiple claims. From a total of 333,289 claims issued for the 89,246 patients with multiple claims, we identified 114,404 AMI episodes according to the algorithm described in the materials and methods section. The final number of total identified discrete AMI admissions was 295,001 (Figure 1). Overall, 53.4% of the episodes involved admission through the emergency department. Male and middle-aged (40 to 69 years) patients were more likely to be admitted through the emergency department. Altogether, 27.6% of AMI patients underwent invasive or surgical procedures during hospitalization. Male and middle-aged (40 to 69 years) patients were more likely to undergo invasive procedures (Table 1).

**Figure 2.**
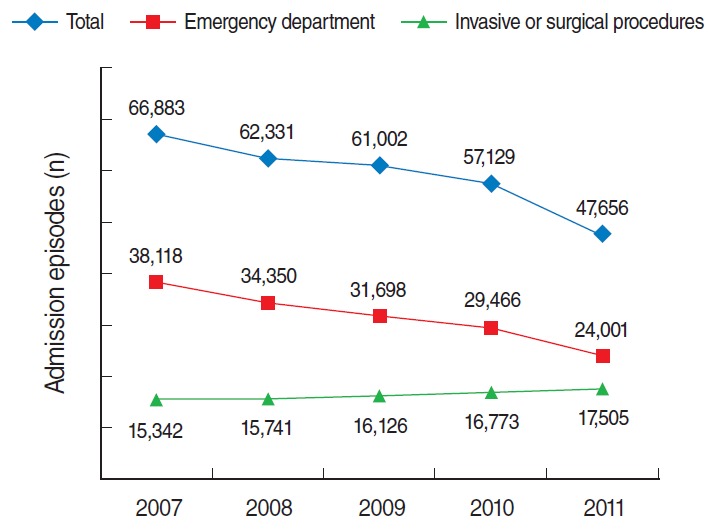
Quantity of total admissions for acute myocardial infarction, admissions through the emergency department, and admissions involving invasive or surgical procedures over time.

**Figure 3.**
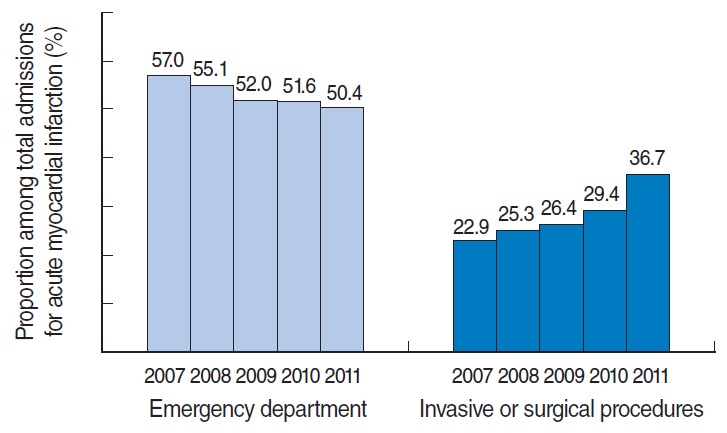
Proportions of admissions through the emergency department and admissions involving invasive or surgical procedures among total admissions for acute myocardial infarction admissions in each year.

The total number of AMI admissions gradually decreased from 66,883 in 2007 to 47,656 in 2011. This trend was consistent across both sexes and all age groups (Table 2). Moreover, the number and proportion of admissions through the emergency department also decreased during the same period. In 2011, only half of all AMI admissions were through the emergency department. In contrast, the number and proportion of patients who underwent invasive or surgical procedures during hospitalization gradually increased. The number of admission episodes that were accompanied by invasive or surgical procedures was 15,342 (22.9% of total admissions) in 2007, but the corresponding number was 17,505 (36.7% of total admissions) in 2011 (Table 3). Figure 2 shows the decreasing trend in the reported numbers of hospital admissions and emergency department visits for AMI, as well as the increasing trend in hospital admissions involving invasive or surgical procedures. Figure 3 shows that the proportion of admissions through the emergency department among all AMI admissions decreased, although the proportion of admissions involving invasive or surgical procedures increased between 2007 and 2011.

## DISCUSSION

Through an analysis of the NHI claims database, we observed that the reported number of AMI admissions decreased from 2007 to 2011. We also observed that the number of emergency department visits for AMI decreased, but the number of AMI admissions involving invasive or surgical procedures increased.

Two studies have previously estimated the incidence of AMI in Korea based on analyses of the NHI claims database. One study concluded that the incidence of AMI increased between 1997 and 2007 [5], while another study reported that the incidence of AMI decreased between 2006 and 2010 [6]. It is inappropriate to directly compare the absolute incidence rates estimated by these two studies, because they used different methods to identify AMI admissions. However, it is still notable that the two studies reported opposite trends in the incidence of AMI, even within a short overlapping period (2006 to 2007). Our study provides at least a partial explanation of these conflicting results. According to other studies that analyzed hospital records, over 90% of patients with AMI were admitted through the emergency department, and over 70% underwent invasive procedures during hospitalization [7,17]. We assume that majority of patients with AMI would visit the emergency departments when the symptoms occurred, and majority of patients would also undergo invasive diagnostic or therapeutic procedures during their hospitalization. In our study, we found that almost half of the reported AMI admissions were not through the emergency department, and that only a small portion of the admissions were accompanied by invasive or surgical procedures. These findings suggest that trends in insurance claims for AMIs may not reflect trends in AMI incidence.

We evaluated trends in total AMI admissions using the corresponding diagnosis codes in the NHI claims database. Diagnoses in the NHI claims database do not perfectly match clinical diagnoses, resulting in misclassification [8]. The decline in reported AMI admissions might be due to the decrease of false positives, as diagnostic techniques have improved and health insurance review has become more ubiquitous [5]. The decreasing numbers of admissions through the emergency department can be partially explained by the decrease of total AMI admissions. However, the decreasing proportion of admissions through the emergency department among total AMI admissions implies that there are other factors in play. The proportion of non-ST-segment elevation myocardial infarction (NSTEMI) among total AMI patients has increased in recent years [18-20]. Patients with NSTEMI are less likely to present with typical chest pain or discomfort than patients with ST-segment elevation myocardial infarction (STEMI) [21]. The increase in NSTEMI patients might be related to the observed decrease in emergency department admissions, as the severity of symptoms affects the utilization of emergency medical services [22].

The use of invasive revascularization strategies is increasing among NSTEMI patients [18]. Therefore, the increasing proportion of NSTEMI cases might be also related to the observed increase in invasive procedures among total AMI admissions [18]. Invasive revascularization strategies have been proven to improve the outcome of AMI patients, in comparison with conservative or thrombolytic therapy [23]. The preference for invasive procedures over non-invasive medical treatment could increase the use of invasive procedures [24]. We also observed a disparity between men and women in the proportion of patients who underwent invasive or surgical procedures. This result is consistent with previous studies, which have reported sex disparities in the distribution of patients who receive revascularization procedures [25]. The causes of these disparities are not yet fully understood, but a lower chance of undergoing revascularization procedures may accompany the high frequency of cardiovascular disease risk factors and atypical symptoms in females [26]. Findings from previous studies and the present study suggest that the relative prevalence of STEMI and NSTEMI has recently changed in Korea [17,19]. An increase in NSTEMI cases may affect the utilization of the emergency department and invasive procedures [18-22]. A simple analysis of the NHI claims database may overestimate the incidence of AMIs. We cannot fully explain why emergency department visits for AMI have decreased while invasive procedures have become increasingly common. Nonetheless, we would point out that an analysis limited to the diagnosis codes in the NHI claims database cannot provide valid information for estimating the incidence of AMIs.

The major advantage of this study is the high coverage level of the NHI [4]. We suggest that the results in this study represent trends in emergency department visits for AMIs and the rate of invasive procedures among AMI admissions in the Korean population. We also developed an algorithm to identify discrete admission episodes based on the date of admission, date of discharge, and length of admission. It is important to distinguish the number of insurance claims and the number of disease episodes, because multiple claims are possible for a single disease event [9]. The discrepancy between insurance claims and disease episodes varies widely depending on the disease in question [8, 27]. Therefore universal algorithms cannot be applied to various kinds of diseases.

This study also has several limitations that should be addressed. First, the NHI claims database cannot identify patients who did not use medical services [6]. However, this underestimation would not severely influence the proportion of emergency department visits or the proportion of patients who underwent invasive procedures. Second, we included coronary angiography in the category of invasive procedures. Since the reasons for undergoing invasive procedures and their results were not available in the NHI claims database, we did not have enough information to determine whether coronary angiography was performed for purely diagnostic purposes or was performed in connection with therapeutic procedures. Therefore, the trend in the number of admission episodes involving invasive procedures may not reflect a real trend in AMI incidence. Third, in our data, the admission route was not recorded for approximately 2.4% of AMI admissions. In addition, no data are available regarding the accuracy of the admission routes recorded in the NHI claims database. Further studies are required to assess the validity and reliability of the NHI claims database. Fourth, we did not have information regarding whether the invasive procedures were performed appropriately. The feasibility of using invasive procedures or a preference for invasive procedures over non-invasive medical therapy might produce a tendency to overuse invasive procedures, but no information regarding this issue was accessible in this study. Finally, including AMI admission episodes coded with secondary diagnoses might have led to overestimating newly developed AMI episodes. In most patients with newly developed AMI, AMI would be recorded as the primary diagnosis. AMI admission episodes coded with secondary diagnoses might include patients with a previous history of AMI. The low proportions of emergency department visits and admissions involving invasive procedures imply this overestimation. However, this overestimation would not affect trends in the utilization of medical resources. Analyzing AMI admission episodes restricted to primary diagnoses is likely to be more appropriate for evaluating medical usage patterns in newly developed cases of AMI. Further studies incorporating information from medical records or laboratory tests could improve the validity of analyses based on the claims database [28].

In conclusion, the total number of NHI claims for AMI admissions decreased between 2007 and 2011. However, AMI admissions accompanied by coronary angiography and/or coronary revascularization procedures increased during the same period. Moreover, unexpectedly small proportions of AMI admissions were through the emergency department or involved invasive procedures. Our findings suggest that simply counting the number of admission claims cannot provide valid information about trends in AMI events. Careful interpretation is required to discern the actual trends in AMI admissions based on an analysis of health insurance claims.

## Figures and Tables

**Table 1.** Characteristics of patients admitted for acute myocardial infarction, 2007-2011

	Total admission episodes		Admissions through emergency department			Admissions with invasive or surgical procedures		
Variables	n	Column (%)	n	%	Proportion of total admission episodes (%)^1^	n	%	Proportion of total admission episodes (%)^1^
Total	295,001	100.0	157,633	100.0	53.4	81,487	100.0	27.6
Male	166,081	56.3	94,244	59.8	56.7	58,355	71.6	35.1
Female	128,920	43.7	63,389	40.2	49.2	23,132	17.9	17.9
Age (yr)								
0-9	341	0.1	153	0.1	44.9	2	0.0	0.6
10-19	2,053	0.8	820	0.5	39.9	4	0.0	0.2
20-29	4,710	1.7	2,171	1.4	46.1	179	0.2	3.8
30-39	11,503	4.9	6,136	3.9	53.3	2,332	2.9	20.3
40-49	31,631	14.2	17,755	11.3	56.1	10,640	13.1	33.6
50-59	50,333	21.9	28,248	17.9	56.1	19,018	23.3	37.8
60-69	64,707	24.0	34,383	21.8	53.1	21,556	26.5	33.3
70-79	80,912	22.5	42,691	27.1	52.8	20,696	25.4	25.6
≥ 80	48,791	10.0	25,276	16.0	51.8	7,060	8.7	14.5

1 Proportion (%) among total admission episodes in each row.

**Table 2.** Number of total admission episodes for acute myocardial infarction by year

Variables	2007	2008	2009	2010	2011
Total	66,883 (100.0)	62,331 (100.0)	61,002 (100.0)	57,129 (100.0)	47,656 (100.0)
Male	37,521 (56.1)	34,814 (55.9)	34,388 (56.4)	32,176 (56.3)	27,182 (57.0)
Female	29,362 (43.9)	27,517 (44.1)	26,614 (43.6)	24,953 (43.7)	20,474 (43.0)
Age (yr)					
0-9	70 (0.1)	76 (0.1 )	82 (0.1)	82 (0.1)	31 (0.1)
10-19	301 (0.5)	375 (0.6)	504 (0.8)	534 (0.9)	339 (0.7)
20-29	971 (1.5)	1,016 (1.6)	1,108 (1.8)	950 (1.7)	665 (1.4)
30-39	2,594 (3.9)	2,560 (4.1)	2,531 (4.1)	2,284 (4.0)	1,534 (3.2)
40-49	7,324 (11.0)	6,848 (11.0)	6,669 (10.9)	6,039 (10.6)	4,751 (10.0)
50-59	11,134 (16.6)	10,478 (16.8)	10,268 (16.8)	9,649 (16.9)	8,804 (18.5)
60-69	15,628 (23.4)	14,018 (22.5)	13,176 (21.6)	12,213 (21.4)	9,672 (20.3)
70-79	18,592 (27.8)	16,952 (27.2)	16,657 (27.3)	15,531 (27.2)	13,180 (27.7)
≥ 80	10,249 (15.3)	10,008 (16.1)	10,007 (16.4)	9,847 (17.2)	8,680 (18.2)

Values are presented as number (%).

**Table 3.** Proportions of admissions through the emergency department and admissions involving invasive or surgical procedures among total admissions for acute myocardial infarction by sex and year

Year	No. of total admissions	Admissions through emergency department		Admissions with invasive or surgical procedures	
		n	% (95% CI)^1^	n	% (95% CI)^1^
Total					
2007	66,883	38,118	57.0 (56.6, 57.4)	15,342	22.9 (22.6, 23.3)
2008	62,331	34,350	55.1 (54.7, 55.5)	15,741	25.3 (24.9, 25.6)
2009	61,002	31,698	52.0 (51.6, 52.4)	16,126	26.4 (26.1,26.8)
2010	57,129	29,466	51.6 (51.2, 52.0)	16,773	29.4 (29.0, 29.7)
2011	47,656	24,001	50.4 (49.9, 50.8)	17,505	36.7 (36.3, 37.2)
Male					
2007	37,521	22,136	59.0 (58.5, 59.5)	10,848	28.9 (28.5, 29.4)
2008	34,814	20,135	57.8 (57.3, 58.4)	11,190	32.1 (31.7, 32.6)
2009	34,388	18,884	54.9 (54.4, 55.4)	11,544	33.6 (33.1,34.1)
2010	32,176	17,928	55.7 (55.2, 56.3)	12,136	37.7 (37.2, 38.2)
2011	27,182	15,161	55.8 (55.2, 56.4)	12,637	46.5 (45.9, 47.1)
Female					
2007	29,362	15,982	54.4 (53.9, 55.0)	4,494	15.3 (14.9, 15.7)
2008	27,517	14,215	51.7 (51.1,52.2)	4,551	16.5 (16.1, 17.0)
2009	26,614	12,814	48.1 (47.5, 48.7)	4,582	17.2 (16.8, 17.7)
2010	24,953	11,538	46.2 (45.6, 46.9)	4,637	18.6 (18.1, 19.1)
2011	20,474	8,840	43.2 (42.5, 43.9)	4,868	23.8 (23.2, 24.4)

CI, confidence interval.

1 Proportion (95% CI) of total admissions for acute myocardial infarction in each year.
